# A randomized trial of a specialist palliative care intervention for patients undergoing surgery for cancer: rationale and design of the Surgery for Cancer with Option of Palliative Care Expert (SCOPE) Trial

**DOI:** 10.1186/s13063-019-3754-0

**Published:** 2019-12-11

**Authors:** Myrick C. Shinall, Aimee Hoskins, Alexander T. Hawkins, Christina Bailey, Alaina Brown, Rajiv Agarwal, Maria C. Duggan, Laura M. Beskow, Vyjeyanthi S. Periyakoil, David F. Penson, Ryan T. Jarrett, Rameela Chandrasekhar, E. Wesley Ely

**Affiliations:** 1 Division of General Surgery, Department of Surgery, Vanderbilt University Medical Center, 1161 21st Avenue South, Room D5203 MCN, Nashville, TN 37232 USA; 2 Critical Illness, Brain Dysfunction, and Survivorship Center, Nashville, TN USA; 3 Section of Palliative Care, Department of Medicine, Vanderbilt University Medical Center, Nashville, TN USA; 4 Center for Biomedical Ethics and Society, Vanderbilt University Medical Center, Nashville, TN USA; 5 Section of Colon & Rectal Surgery, Division of General Surgery, Vanderbilt University Medical Center, Nashville, TN USA; 6 Division of Surgical Oncology, Department of Surgery, Vanderbilt University Medical Center, Nashville, TN USA; 7 Division of GYN Oncology, Department of OB/GYN, Vanderbilt University Medical Center, Nashville, TN USA; 8 Vanderbilt-Ingram Cancer Center, Division of Hematology and Oncology, Department of Medicine, Vanderbilt University Medical Center, Nashville, TN USA; 9 Geriatrics Research, Education, and Clinical Center, Veterans Affairs Tennessee Valley Health System, Nashville, TN USA; 10 Division of Geriatric Medicine, Department of Medicine, Vanderbilt University Medical Center, Nashville, TN USA; 11 Stanford University School of Medicine, Palo Alto, CA USA; 12 Department of Urology, Vanderbilt University Medical Center, Nashville, TN USA; 13 Department of Biostatistics, Vanderbilt University School of Medicine, Nashville, TN USA; 14 Division of Allergy, Pulmonary, and Critical Care Medicine, Department of Medicine, Vanderbilt University Medical Center, Nashville, TN USA

**Keywords:** Palliative care, Surgical oncology, Cancer, Randomized controlled trials, Trial protocol

## Abstract

**Background:**

In medical oncology settings, early specialist palliative care interventions have demonstrated improvements in patient quality of life and survival compared with usual oncologic care. However, the effect of early specialist palliative care interventions in surgical oncology settings is not well studied.

**Methods:**

The Surgery for Cancer with Option for Palliative Care Expert (SCOPE) Trial is a single-center, prospective, single-blind, randomized controlled trial of a specialist palliative care intervention for cancer patients undergoing non-palliative surgery. It will enroll 236 patients scheduled for major abdominal operations for malignancy, who will be randomized 1:1 at enrollment to receive usual care (control arm) or specialist palliative care consultation (intervention arm). Intervention arm patients will receive consultations from a palliative care specialist (physician or nurse practitioner) preoperatively and postoperatively. The primary outcome is physical and functional wellbeing at 90 days postoperatively. Secondary outcomes are quality of life at 90 days postoperatively, posttraumatic stress disorder symptoms at 180 days postoperatively, days alive at home without an emergency room visit in the first 90 postoperative days, and overall survival at 1 year postoperatively. Participants will be followed for 3 years after surgery for exploratory analyses of their ongoing quality of life, healthcare utilization, and mortality.

**Discussion:**

SCOPE is an ongoing randomized controlled trial evaluating specialist palliative care interventions for cancer patients undergoing non-palliative oncologic surgery. Findings from the study will inform ways to identify and improve care of surgical patients who will likely benefit from specialist palliative care services.

**Trial registration:**

ClinicalTrials.gov Identifier: NCT03436290

First Registered: 16 February 2018

Enrollment Began: 1 March 2018

Last Update: 20 December 2018

## Background

In the past decade, research on early palliative care interventions for life-threatening diseases, especially cancer, has flourished. Palliative care interventions have both decreased utilization of aggressive care at the end of life and increased hospice enrollment and the duration of time under hospice care [1, 2]. Palliative care interventions have also been shown to have beneficial and durable effects before the end of life by improving physical functioning, quality of life (QoL), satisfaction with medical care, and symptom burden [1–6]. In patients with advanced cancer, trials have additionally demonstrated a survival benefit with the early initiation of palliative care [1, 3].

Trials of early palliative care have generally enrolled patients with incurable diseases, such as metastatic cancer or end-stage organ failure. There have been limited data to suggest that early palliative care initiation may be beneficial for patients with hematologic malignancies undergoing treatment with curative intent [7, 8]. Palliative care interventions might therefore benefit patients undergoing other curative, yet potentially morbid, therapies for cancer, such as surgery.

One previous trial studied the effect of a palliative care intervention for patients with advanced cancer undergoing surgery, but not necessarily resections with curative intent [9]. In order to test the efficacy of palliative care in patients undergoing potentially curative resections for abdominal malignancies, the Surgery for Cancer with Option of Palliative Care Expert (SCOPE) Trial was designed.

The SCOPE Trial aims to test the hypothesis that a specialist palliative care intervention will improve the functional and physical QoL of patients undergoing surgery for selected abdominal malignancies. Since improved functional and physical QoL is likely related to better overall well-being and health, the SCOPE Trial will also investigate the association of the intervention with other patient-reported outcomes as well as with utilization of healthcare resources.

## Methods/design

### IRB approval

The SCOPE Trial was approved by the Vanderbilt University Medical Center (VUMC) Institutional Review Board (IRB). Any protocol modifications will be approved by the IRB, and the study information in ClinicalTrials.gov will be updated if the protocol modification affects these reported elements. SCOPE is a prospective, single-center, single-blind, randomized controlled trial designed to test the effect of a specialist palliative care intervention versus routine care for patients undergoing surgery for abdominal malignancies. The study is single-blind in that outcomes assessors remain unaware of treatment assignment, but patients, providers, and other study staff are by necessity aware of treatment assignment.

### Population/setting

Participants are recruited from four surgical clinics at VUMC: general surgical oncology, colorectal surgery, gynecologic oncology, and urologic oncology. Study staff screen daily clinic schedules to identify patients being evaluated for selected major abdominal operations for known or suspected malignancy (see Inclusion criteria). Study personnel approach patients after they have consented to one of the included operations to inform them about the study and obtain informed consent. Patients have the option to consider participation after they leave the clinic and sign a consent form electronically.

#### Inclusion criteria

Patients are eligible to participate if they are 18 years of age or older and scheduled for one of the following operations with intent to provide cure or durable oncologic control of a known or suspected malignancy:

1. Total or partial gastrectomy requiring anastomosis
2. Total or partial pancreatectomy
3. Partial hepatectomy
4. Colectomy or proctectomy if one of the following conditions is also met:
  - Patient age is 65 years or older
  - Disease is metastatic (i.e., oligometastatic disease with plan for concurrent or subsequent metastasectomy)
  - Disease is locally invasive requiring extensive resection (i.e., disease invades into other viscera or abdominopelvic wall)
5. Radical cystectomy
6. Pelvic exenteration
7. Abdominal debulking for ovarian or endometrial carcinoma
8. Cytoreductive surgery and hyperthermic intraperitoneal chemotherapy

The operations were chosen based on their associated morbidity and high rates of mortality from the underlying malignancies treated by these operations. Because colorectal resections are generally better tolerated than the other included operations and surgically resected colorectal cancer has a relatively favorable diagnosis, additional patient or tumor characteristics were included to define a higher-risk population for colorectal resections.

#### Exclusion criteria

We will not enroll patients who:

1. Do not speak English
2. Live > 150 miles away from Vanderbilt and do not visit the Nashville area regularly
3. Have no telephone or are otherwise unwilling/unable to complete follow-ups
4. Are prisoners
5. Are currently enrolled in a study that does not allow co-enrollment or that uses a non-pharmacologic, non-procedural intervention directed at surgical or cancer care
6. Are deaf
7. Have severe prior cognitive or neurodegenerative disorders that prevent patients from living independently at baseline
8. Have one of the following barriers to consent:
  - Attending surgeon refusal
  - Patient refusal
  - Period of time between screening patient and time of operation does not allow preoperative outpatient palliative care visit
9. Are already receiving services from a palliative care specialist

### Baseline assessment and randomization

**Fig. 1:**
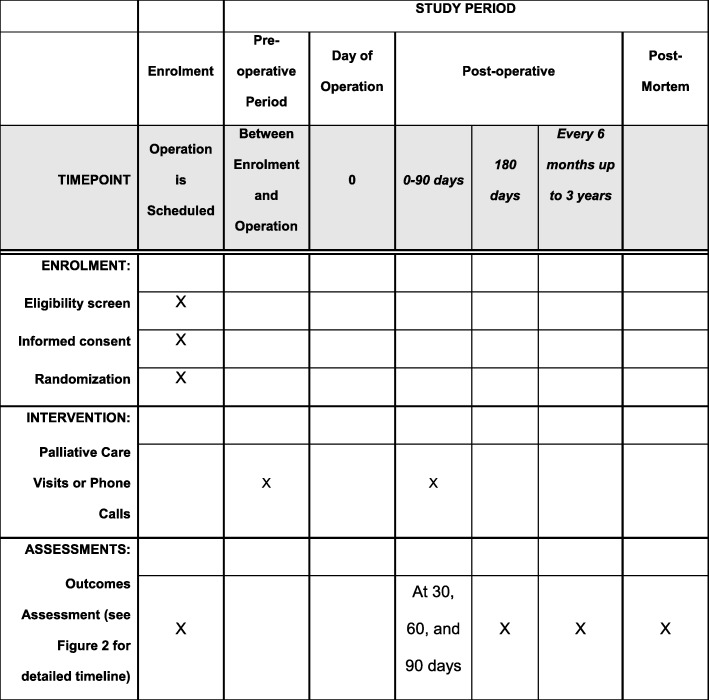
Timeline of study events

**Fig. 2:**
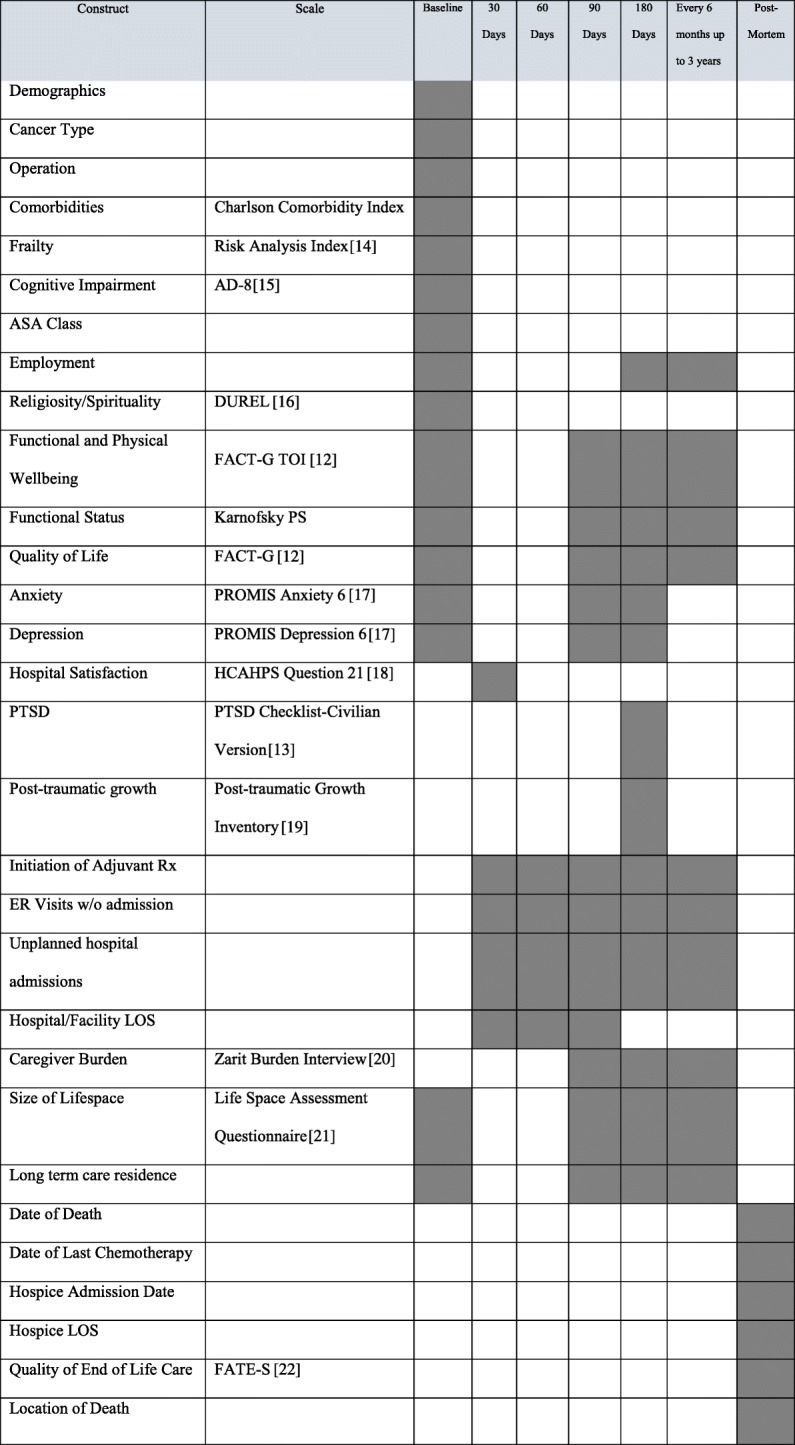
Schedule of baseline/outcomes data collection

The timeline of study events is given in Fig. 1. Once patients provide consent, study staff gather basic demographic information and administer several validated survey instruments to collect baseline data for each participant (Fig. 2). Additionally, the patient identifies a caregiver who will be the participant for caregiver evaluation and will be the contact person in later phases of the study if the patient is unable to respond for him or herself. After these baseline demographic, clinical, and psychometric data are obtained, randomization occurs. To maintain group balance among types of malignancy, a critical determinant of outcome, randomization is stratified by surgical specialty (general surgical oncology and colorectal surgery in one stratum, urologic and gynecologic surgery in the other). We randomize patients meeting all eligibility requirements in a 1:1 ratio to specialist palliative care (intervention) or usual care (control) using a computer-generated randomization scheme using a permuted block design, stratified by surgical specialty. The randomization scheme was created by the trial’s primary biostatistician and has been directly uploaded into REDCap’s randomization module [10].

### Control group (usual care)

After consultation with their surgeon with a decision to operate, patients have a preoperative evaluation by an anesthesia provider and any other consultations deemed necessary. The surgical team provides postoperative follow-up in the hospital, in some cases with assistance from an anesthesia pain team. After hospital discharge, patients generally have one follow-up visit with their surgeon 3–6 weeks postoperatively, with more visits if patients have persistent problems related to their surgery (e.g., wound problems). If the patient needs further chemotherapy or radiotherapy, they generally see those providers (who may or may not be at VUMC) in the ensuing months. If no further cancer-directed therapy is needed, surveillance (either by the surgeon or oncologist) continues, usually at annual or semi-annual intervals. At our institution, routine practice does not include referral to a palliative care provider unless the patient has very difficult-to-manage symptoms or is near the end of life. Retrospective review of patients undergoing surgery for the malignancies in the SCOPE Trial at VUMC revealed that fewer than 10% received a palliative care consultation at any point in their care. In almost all cases, palliative care consultation occurred after surgery. Nevertheless, control group patients can receive palliative care consultations if their clinical providers desire. Palliative care consultations for control patients will be monitored by electronic medical record review, and number and timing of palliative care consultations will be recorded.

### Intervention group (specialist palliative care)

Patients assigned to the intervention group receive all the routine care described above for the control patients. Additionally, after randomization, intervention arm patients are immediately scheduled for a preoperative outpatient palliative care consultation by a physician or nurse practitioner on the Vanderbilt palliative care team. This initial consultation has five areas of focus:

1. Palliative care is introduced as a specialty focused on improving the QoL of patients with serious illnesses, and the provider discusses how specialist palliative care will be integrated into the patients’ overall care.
2. The provider assesses the goals of care by eliciting the patients’ values, what matters most to them, and their future priorities for treatment.
3. The provider assesses for any symptoms that might impair patients’ recovery from the operation (e.g., lack of energy from cancer treatments) and makes recommendations for management.
4. The provider asks about the patients’ interest in advance care planning and helps them discuss and document their advance directives.
5. The provider assesses psychosocial or spiritual stressors in the patients’ lives and provides counseling or, if necessary, referral to an appropriate professional (e.g., chaplain, social worker, mental health professional).

If the patient is unable to come to an in-person visit with a palliative care provider, this consultation is conducted by telephone. As a measure of intervention fidelity, study staff assess the completion of these elements as recorded in the medical record. The preoperative palliative care consultation is arranged according to each patient’s schedule and convenience, ideally coordinated with his or her standard presurgical planning. We have ensured that these intervention visits do not delay or interfere with each patient’s scheduled surgical date.

Patients in the intervention group also receive an inpatient palliative care consultation on postoperative Day 1. The palliative care team follows these patients with at least two visits per week of admission. During these visits, palliative care providers assess and make recommendations on seven patient issues: (1) pain, (2) nausea/vomiting, (3) constipation/ileus, (4) sleep disturbance, (5) delirium, (6) impaired mobility, and (7) psychosocial/spiritual distress. Study staff also track completion of these assessments recorded in the medical record as another measure of intervention fidelity.

During the 90 postoperative days, intervention patients are scheduled for three outpatient palliative care visits. If intervention patients cannot be present for any in-person visit, a telephone call follow-up with a palliative care provider is conducted. Because the course of these patients is variable, a prescriptive list of elements for this aspect of the intervention was not developed. Instead, providers use their expert judgment to assess and address these patients’ palliative care needs. After 90 days, intervention patients continue to see their outpatient palliative care provider at a frequency determined by the provider’s assessment of their needs. Additionally, during the 3-year follow-up period, intervention patients receive an inpatient palliative care consultation whenever they are admitted to VUMC, with palliative care providers using their judgment to address any unmet needs or symptoms. Study staff monitor the study census daily to alert providers if any intervention patients have been admitted to VUMC. Intervention patients can decide at any time that they would like to stop seeing palliative care providers. All palliative care services are billed to the patients’ insurance provider and reimbursed as routine clinical services.

### Data collection

Enrolled patients are evaluated at study enrollment, at 30, 60, 90, and 180 days postoperatively, and then every 6 months thereafter for the 3-year follow-up period. Data collection at study enrollment occurs either in person or by telephone (if the electronic consent option is used) by a study nurse before the patient is randomized. Postoperative assessments are conducted by telephone by a different outcomes assessor who remains blinded to treatment assignment. Beginning at the 90-day postoperative assessment, the patient’s caregiver will be contacted to assess caregiver burden at the same time points as the patient assessments.

Study staff will monitor for patient deaths through a combination of medical records scanning and monitoring obituaries online. When it is determined that a participant has died, the study personnel will contact the patient’s designated caregiver to determine the end-of-life care utilization: whether and when the patient enrolled in a hospice, when the patient’s last dose of tumor-directed systemic therapy occurred, hospital admissions and ER visits in the last month of life, and where the patient died. For decedents, a quality of death and dying survey will be administered to the caregiver.

Data collected at each assessment is shown in Fig. 2. The palliative care intervention is impossible to mask from participants, and at least some study staff must be aware of intervention assignment in order to schedule palliative care appointments and monitor intervention compliance. To reduce possible bias, all outcomes data are collected by a separate, blinded outcomes assessor. The REDCap database has been partitioned to prevent the outcomes assessor from inadvertently becoming unblinded, and non-blinded study staff make strict efforts not to reveal group allocations to the blinded outcomes assessor.

### Outcomes

The primary outcome of the SCOPE Trial is functional and physical QoL at 90 days after surgery as measured by the Trial Outcome Index (TOI), which consists of the Physical and Functional Wellbeing Subscales of the Functional Assessment of Cancer Therapy-General (FACT-G). Prior studies of palliative care interventions have shown benefits in patient-reported psychosocial outcomes (e.g. QoL, mood, posttraumatic stress disorder [PTSD]) [1, 3, 7, 8, 11] and in more traditional physical outcomes of disease control (e.g., survival, healthcare utilization) [1, 3]. The FACT-G TOI provides an outcome that bridges both the physical and psychosocial domains, and so was chosen as the trial’s primary outcome, a decision bolstered by the TOI’s use as the primary outcome in one of the landmark studies of early palliative care in lung cancer patients [1]. The TOI is generally more sensitive to change than the FACT-G total score, and so the TOI is frequently used as an endpoint in trials of therapeutic interventions [12]. Overall QoL and PTSD were judged to be two patient- reported outcomes that the intervention was likely to benefit and that clinicians would find compelling. Similarly, we also judged survival and postoperative healthcare utilization to be potentially improved by the intervention, and improvements in these outcomes would persuade clinicians to incorporate specialist palliative care in practice. We therefore chose these four secondary outcomes:

1. Overall QoL at 90 days as measured by the total score on the FACT-G [12]
2. Days alive at home without an emergency room (ER) visit during first 90 days postoperatively
3. PTSD symptoms at 180 days as measured by the PTSD Checklist-Civilian Version [13]
4. Overall survival at 1 year

This list of primary and secondary outcomes, however, does not exhaust the possible benefits of early palliative care interventions. It may be that the benefits of palliative care consultation are only evident over longer time frames or at the end of life, or that other patient-reported outcomes are most improved by the intervention. This study could not be powered to reliably detect all these possible effects, but a number of exploratory outcomes are included that will inform choice of outcomes for future studies. These exploratory outcomes include some of the same measures that constitute the primary and secondary outcomes, but at different time points, as well as different sets of measures that assess survivorship and end-of-life care (see Fig. 2; [14–22]). All baseline and outcomes data are collected orally by an assessor either in person or over the telephone, marked on a paper form, and then entered into the REDCap database with the hard copies of the forms preserved. In addition, an ancillary study is planned to conduct semi-structured interviews with a subset of the participants in both the intervention and control arms to assess their perceptions of their met and unmet needs during their care.

### Sample size calculation and statistical analysis

Previous studies have demonstrated a moderate effect (effect size approximately 0.4) of early palliative care on TOI, the primary outcome of the SCOPE Trial [1]. Assuming a type I error rate of 5% and a common standard deviation of 9 in the FACT-G TOI score and 18.1 in the FACT-G total score in each group [12], enrolling 98 participants in each group (total *N* = 196) would provide at least 80% power to detect a change of 3.6 points for TOI and 7.24 for total FACT-G (an effect size of 0.4). Minimally important difference for the total FACT-G is estimated to be 3–7 points and 2–3 points for each subscale [12], so the trial should be adequately powered to detect a clinically meaningful difference. The sample size computations were performed assuming a normal distribution for the primary outcome and using two-sample *t* test. Allowing for 20% loss to follow-up (we expect mortality to be very low at 90 days), we plan to enroll 236 patients to ensure an adequately powered study. Based on the mix of malignancies included in the study, we expect median survival to be approximately 36 months, so a substantial portion of patients should be alive at later time points, which will enable exploratory analyses with the long-term outcomes.

Closer to the completion of the trial, the investigators and biostatisticians will develop a comprehensive statistical analysis plan (SAP) that will be made publicly available and will be time-stamped. The SAP will describe the analytic strategies used for the study and any prespecified subgroup analyses. The SAP will be completed while the biostatisticians remain blinded to the participants’ group assignment, and any subsequent changes will be publicly noted to have occurred after the blind was broken. In developing our SAP, we will be ready to adopt novel statistical methods that are developed while the trial is ongoing. In what follows, we present a tentative outline for the approach that we will develop in detail in the SAP.

The demographic and clinical characteristics of patients will be described using descriptive statistics. For continuous variables, median and interquartile range will be used while categorical variables will be described using frequency (percentage).

For our primary analysis, we will perform multivariable regression to adjust for a priori-selected potential confounders, including age, frailty, cancer type, insurance status, education level, and degree of religious involvement. We will choose the type of multiple regression by carefully examining the distribution of the data. In the case of non-normally distributed outcomes, we will use a multivariable proportional odds regression model. For normally distributed outcomes, a linear regression model will be used. Cox proportional hazards regression will be used to analyze the adjusted effect of intervention on time-to-event outcomes such as survival, with censoring as appropriate based on the outcome and time point (for instance, the analysis of overall survival at 1 year will have censoring at 1 year for patients who have not died).

For all primary analyses, we will adhere to intention-to-treat principles. Missing data will be imputed using standard model-based imputation methods, i.e., regression imputation where a model is fitted on the observed data and subsequently used to generate imputations for the missing values. Imputation of data will only be performed for baseline characteristics and not for outcomes, and the covariates will include only those obtained at baseline. The SAP will provide further details on the plans for missing data imputation. Non-linear effects of continuous variables will be fit using restricted cubic splines, and modern regression model building techniques will be used [23]. For long-term outcomes, missing data are common due to death and loss to follow-up. We will deal with this potential bias by estimating the principal stratum causal effect (i.e., survivor average causal effect) defined as the average causal effect among participants who would survive under either treatment [24]. Since the analysis of survivors with assessments may be susceptible to survivor bias, we will use an unadjusted composite endpoint approach as described by Lachin [25], where the composite endpoint will be defined as days between surgery and death if the patient dies prior to assessment, but if the patient survives and is successfully assessed, the outcome will be days between surgery and the assessment plus the assessment score. All covariates included in the adjusted models will be selected a priori and the model complexity will be based on the general rule that a model must fit no more than m/10 parameters to allow for proper multivariable analysis and to be generalizable to future patients, where m is the effective sample size [23].

Graphical techniques will be used to perform model diagnostics and evaluate assumptions. Multicollinearity will be assessed using variance inflation factors and in the event of highly collinear variables, principal component analysis will be used. All models will be validated using the bootstrap internal validation approach and the cross-validation approach. No adjustment will be made for multiple comparisons when examining secondary a priori-defined outcomes [26, 27]. Caution will be exercised in the interpretation of results by noting the number of nominally significant tests that would be expected to occur by chance alone [28].

Secondary and subgroup analyses will be prespecified and fully described in the SAP, and at the time of drafting it we will conduct a further review of the literatureto determine which prespecified subgroups are important to study. To alleviate issues caused by multiple testing, our planned subgroup analysis will purely be exploratory in nature and used to inform the design of future studies. Caution will be exercised in the interpretation of results by noting the number of nominally significant tests that would be expected to occur by chance alone [28]. *P* values will not be provided, instead effect sizes will be quantified, and confidence intervals will be provided. Results of subgroup analysis will be graphically illustrated. Currently, we plan to conduct exploratory analyses of the interaction of baseline age, frailty, and cognitive impairment with group assignment because older patients or those with frailty or cognitive impairment may be at increased risk for adverse outcomes and therefore more likely to benefit from the intervention. Such results could inform the design of future studies. Since the control group may receive additional palliative care consults at providers’ discretion and some patients in the intervention group may receive fewer visits than others due to missed appointments, palliative care consults for both groups will be monitored and described. Additionally, sensitivity analysis will be considered where dose of palliative care (i.e., number of visits/telephone calls completed) will be considered as an interaction term with treatment group. In these sensitivity analyses, palliative care will be included as a time-varying covariate. The details of these sensitivity analyses will be described in the SAP.

### Strategies to maximize recruitment, retention, and intervention compliance

Study personnel are physically present in the enrolling clinics’ physician work rooms as much as possible when potentially eligible patients have visits and ask permission to approach patients whenever they find out a patient is having an eligible operation. In the consent process, potential participants are informed that they will receive a USD 50 gift card if they enroll and complete the 3-month assessments. Participants randomized to the intervention arm will be given an additional USD 50 gift card if they come to an in-person palliative care visit preoperatively and a third USD 50 gift card if they come to at least one in-person palliative care clinic visit after hospital discharge.

Study staff monitor recruitment, retention, and completion of outcomes assessment via a computerized dashboard linked to the REDCap database. As data accumulate, they are periodically imported into R statistical software to calculate updated weekly screening, enrollment, and exclusion counts. Patients identified as eligible for follow-ups are tracked to observe whether or not follow-up reports have been completed. Patient withdrawals and deaths are similarly monitored, and a postmortem follow-up rate is calculated. Any patients with missing IDs or operation dates are listed for further investigation, if necessary. These results are succinctly and dynamically displayed through the use of an R Shiny Dashboard, produced by RStudio, which allows the user to interact with and explore summaries of the data to date. This provides near real-time information allowing the investigators to monitor trends in enrollment and follow-up and to identify problems as they occur.

The study staff monitor the medical records of all intervention patients who have not yet reached 90 days postoperatively, and make sure that palliative care visits or telephone calls are scheduled appropriately. When a scheduled outpatient palliative care visit or telephone call does not occur, study staff contact the patient to determine whether they can reschedule. Study staff also monitor the hospital census daily to determine if any intervention patients have been admitted or undergone an operation, and if so, they contact clinical personnel to make sure an inpatient palliative care visit occurs.

### Data management and monitoring

During all study phases, all data will be entered into electronic case report forms (eCRFs) in a secured password-protected database. Copyrighted forms will be used when required. This study will utilize REDCap for data collection, transmission, and storage. All study data will be entered via a password-protected REDCap database website [10]. To ensure data are accurately and completely collected during the SCOPE trial, study personnel will assure that the study protocol is being followed and that changes to the protocol have been approved by the IRB. Also, the study personnel will periodically review study records to determine whether data collected is accurate, complete, and current. Data integrity will be monitored at weekly meetings between the principal investigator (PI) and study staff and through periodic audits of records by study staff different than the one who entered the data to ensure accuracy and completeness.

The study team will provide the first level of data and safety monitoring. This team includes the primary study nurse, the physician PI, and a faculty biostatistician. The PI and study nurse meet at least every week to review patient progress. The study team’s experienced biostatistician monitors data to assure data accuracy. Because of the low-risk nature of the intervention, a data and safety monitoring board was not created.

The PI and study nurse have primary responsibility for insuring scientific integrity and patient safety, monitoring the occurrence of adverse events (AEs), and evaluating impact. In their weekly meetings the PI and study nurse review ongoing protocol compliance. We do not anticipate that palliative care services or participation in this study will directly result in any AEs. Patients will not be exposed to investigational drugs or devices as part of this study. The palliative care intervention contains standard-of-care procedures for patients receiving palliative care services at VUMC. The only additional process is the collection of patient-reported outcomes by study personnel.

However, if any AEs are identified to be possibly or probably associated with the study procedures (e.g., increased anxiety from completion of QoL questionnaires), they will be documented within the study record and reported to the PI and IRB per IRB guidelines. Unanticipated problems (unexpected, possibly related events that may place participants or others at a greater risk of harm) will be reported to the PI and IRB as soon as possible, and within 7 calendar days of study staff becoming aware of the event or problem.

### Dissemination plan

The SCOPE Trial has been registered with ClinicalTrials.gov. Within 12 months of completion of the trial, the ClinicalTrials.gov site will be updated to include summary results. Additionally, the investigators will seek to disseminate the results of the study through peer-reviewed literature. Authorship on publications will be on the basis of substantive contribution to the study in accordance with recommendations from the International Committee of Medical Journal Editors. The full study protocol will be made available with publication of results. VUMC has internal policies to ensure that clinical trials registration and results reporting occur in compliance with policy requirements; the investigators will adhere to these internal policies to ensure compliance.

## Discussion

The SCOPE Trial will assess the effect of preoperative, perioperative, and postoperative specialist palliative care for patients undergoing major abdominal operations for cancer. Efficacy of the intervention will be defined primarily by increased physical and functional QoL at 90 days postoperatively in the intervention group compared to control group patients receiving usual care. Improvements in the secondary outcomes in the intervention versus the control group would also provide evidence of efficacy of the intervention. Additionally, the follow-up conducted over 3 years will provide a wealth of information on various outcomes that will be analyzed in an exploratory fashion.

Trial results may be biased toward the null if control patients receive palliative care consultations. The eligible patient population, however, rarely receives palliative care consultations as part of routine clinical practice. Study staff monitor control group use of palliative care carefully. If the staff discover substantial numbers of control patients receiving palliative care consultations, we will discuss with the involved surgeons whether their practice has changed and whether or not to continue enrolling patients from their clinics. After a year of enrollment, we have detected no such changes.

In addition to this possibility of bias from control patients receiving palliative care, there are other limitations to this study that will temper interpretation of a null result. It is possible that specialist palliative care benefits patients undergoing surgery for cancer in ways not captured by the outcomes for this study. However, in a recent meta-analysis of randomized trials of palliative care interventions, improved QoL of intervention patients compared to control patients at 1- to 3-month follow-up was one of the most consistently observed results across studies [29]. The primary endpoint of the SCOPE Trial is the TOI (a subset of the FACT-G QoL instrument) at 3 months postoperatively and one key secondary endpoint is the FACT-G total score at 3 months postoperatively, so if the intervention benefits patients, it seems likely one of these endpoints would be affected. We have also attempted to address this limitation by collecting a large number of exploratory outcomes that could detect possible benefits of the intervention not reflected in the primary or secondary endpoints. Results on these exploratory outcomes could then inform further trials dedicated to testing the effect of a specialist palliative care intervention on these outcomes.

Another limitation relevant to the interpretation of a null result is the heterogeneity of the patient population in type of cancer and operation. The included operations have different trajectories of recovery, and the different malignancies have different treatment paradigms and survival rates, which will add variability to the outcomes measurements. A meaningful effect of the intervention could be overwhelmed by the variability in this heterogeneous patient population and thus remain undetected. To mitigate this liability, we will use adjusted analyses as our primary analysis of the outcomes to improve the precision of the estimates of the effect of the intervention and control for the variability inherent in a study enrolling patients with different diseases.

A relevant limitation if the study demonstrates a difference between the control and outcomes group is that the study will provide no means of determining how the intervention achieved that result. Since there is no attention control group in the study, it is possible that any differences between intervention and control groups are simply the result of the intervention patients having more contact with clinicians rather than something specific about the nature of the palliative care intervention. We chose not to have an attention control group so that our control group reflects current clinical practice. If the study shows a positive result, further studies comparing specialist palliative care interventions to other less intensive forms of palliative care or attention controls could be conducted. Our planned ancillary study using semi-structured interviews with participants will help inform our understanding of how the intervention may have affected outcomes.

As a single-center trial, results of the study may not translate into other settings. We have attempted to structure the intervention and standardize the contents of the palliative care discussions as much as possible. Nevertheless, all patient-provider interactions are inherently fluid and require the providers to make judgments and react to what the patients tell them, so the intervention cannot be completely structured by a protocol. Results of the trial may therefore reflect our palliative care providers’ particular styles within the logistical structures of our institution, which may not be present elsewhere.

Despite these limitations, the SCOPE Trial will provide important information about whether a specialist palliative care intervention can improve outcomes for patients undergoing major abdominal operations for malignancy. As data for the benefits of palliative care in medical oncology settings accumulates, it is crucial to determine whether specialist palliative care shows similar benefits in the surgical oncology setting. If the study shows the intervention to be beneficial, clinicians could expand specialist palliative care programs to include these patients. If the study shows no benefit of the intervention, this knowledge will help us direct scarce palliative care resources to more fruitful populations.

## Trial status

Protocol Version: 1.03 (March 18, 2019)

Recruitment began: March 1, 2018

Approximate date of recruitment completion: December 1, 2021

## Data Availability

Not applicable

## Abbreviations

AD8: Alzheimer disease-8
AE: Adverse event
ASA: American Society of Anesthesiologists,
DUREL: Duke University Religion Index
eCRF: Electronic case report form
ER: Emergency room
FACT-G: Functional Assessment of Cancer Therapy-General
FATE-S: Family Assessment of Treatment at End of Life Short Form,
HCAHPS: Hospital Consumer Assessment of Healthcare Providers and Systems
IRB: Institutional Review Board
LOS: Length of stay
PI: Principal investigator
PROMIS: Patient-Reported Outcomes Measurement Information System
PS: Performance status
PTSD: Posttraumatic stress disorder
QoL: Quality of life
Rx: Prescription
SAP: Statistical analysis plan
SCOPE: Surgery for Cancer with Option for Palliative Care Expert
TOI: Trial Outcome Index
VUMC: Vanderbilt University Medical Center
